# Congenital aneurysmal right coronary artery with a fistula to the left atrium in an adult

**DOI:** 10.1186/s13019-019-0854-6

**Published:** 2019-02-08

**Authors:** Neerod Kumar Jha, Laszlo Kiraly, Nishant Shah, Arif Al Mulla, Bassem Mora

**Affiliations:** Institute of Cardiac Sciences, PO BOX 51900 Sheikh Khalifa Medical City, Abu Dhabi, United Arab Emirates

**Keywords:** Fistula, Aneurysm, Coronary, Artery, Atrium, Cardiac, Surgery, Percutaneous, Intervention

## Abstract

**Background:**

Congenital coronary artery fistula in association with aneurysm of the involved coronary artery in adults is rare. Moreover, the right coronary artery- left atrial fistula is also uncommon. Most of the cases are asymptomatic. However, symptomatic patients need therapeutic interventions. The potential complications associated with this anomaly are life-threatening, therefore, there is a need to explore more on differential diagnosis, investigations, management strategies and prevention of complications.

**Case presentation:**

We present herewith a 26-year-old male patient with symptoms of chest pain and dyspnea. He was diagnosed with aneurysmal dilatation of the right coronary artery in its entire course which terminated as a fistulous communication into the left atrium. The closure of the fistula was done using autologous pericardial patch under cardiopulmonary bypass. Currently, the patient is being followed up after surgery and receiving anticoagulants.

**Conclusion:**

The advancement in the diagnostic imaging modalities have made it possible to find similar abnormalities more frequently. Due to rare nature of this anomaly, there is a need to explore and discuss management strategies that include medical management, surgical intervention or percutaneous interventions for a successful outcome.

## Background

Congenital coronary artery fistula (CAF) in association with aneurysm of the involved coronary artery in adults is rare [1–7]. The coronary artery fistula is anomalous termination of the coronary artery into the cardiac chamber, coronary sinus, pulmonary artery or pulmonary vein. The left atrium is involved in approximately 5% patients with CAF [1]. In majority of the patients, it is asymptomatic and an incidental finding. However, progress in the diagnostic imaging modalities have made it possible to find such abnormalities more frequently even in asymptomatic patients. We present herewith an adult male who presented with chest discomfort and congestive heart failure. He underwent surgical correction of a large right coronary artery- left atrial fistula.

## Case presentation

A 26-years-old male patient presented with history of occasional mild central chest discomfort and mild dyspnea on exertion of 8-months duration. During last 4 months, the dyspnea (NYHA III) was progressing rapidly. He was receiving medications for congestive heart failure.

**Fig. 1:**
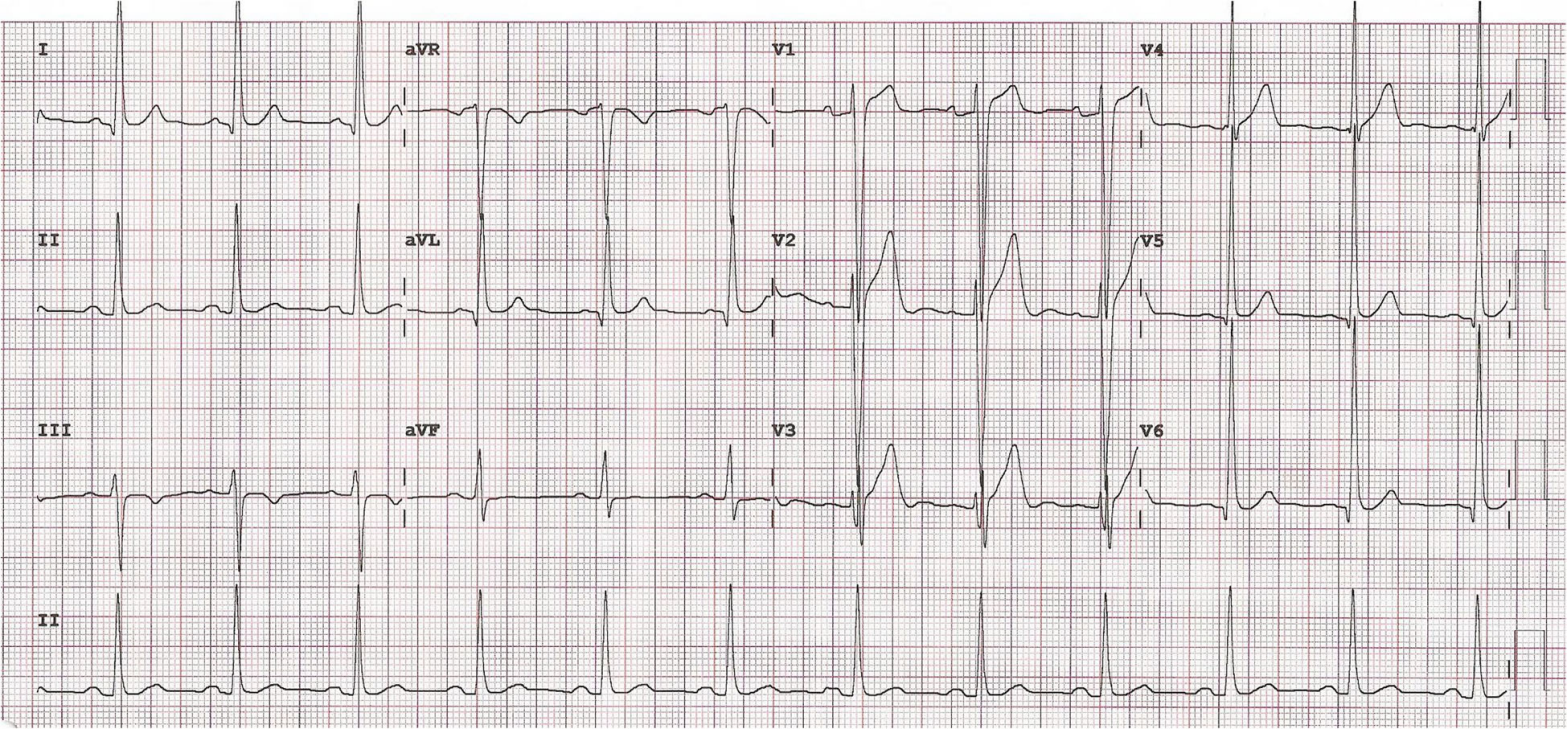
Electrocardiogram showing ‘ST’ changes in the anterior leads and evidence of left ventricular enlargement

There was a continuous murmur over the precordium. His blood pressure in the right-arm was 135/54 mmHg. There was no systemic or pulmonary edema. Electrocardiogram was consistent with sinus rhythm, mild ‘ST-elevation’ in the ‘V1-V3’ leads and left ventricular enlargement (Fig. 1). However, there was no evidence of myocardial ischemia.

**Fig. 2:**
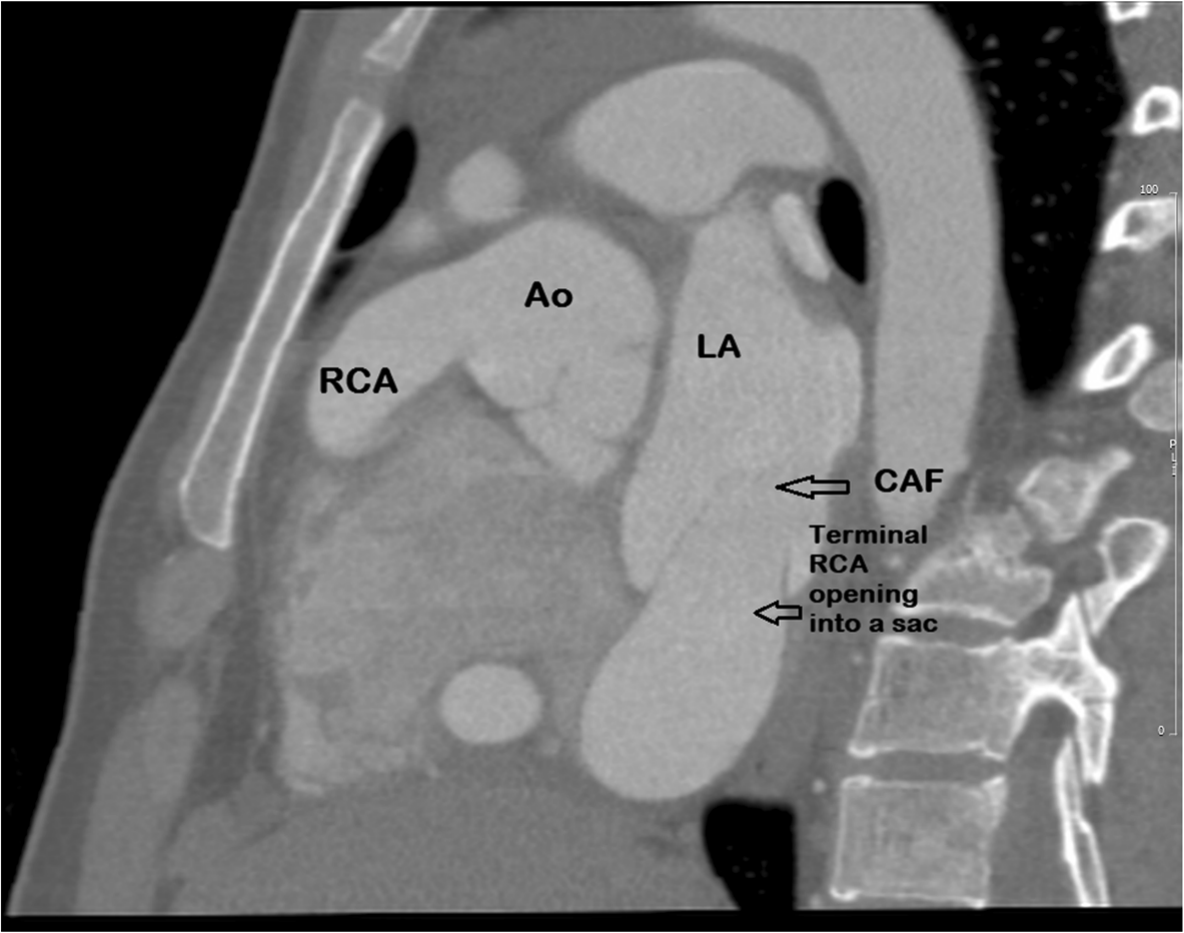
A CT angiogram (sagittal view) showing origin of the dilated coronary artery from the ascending aorta. Ao- Ascending aorta, RCA-Right coronary artery, LA-Left atrium, CAF- Coronary artery fistula

**Fig. 3:**
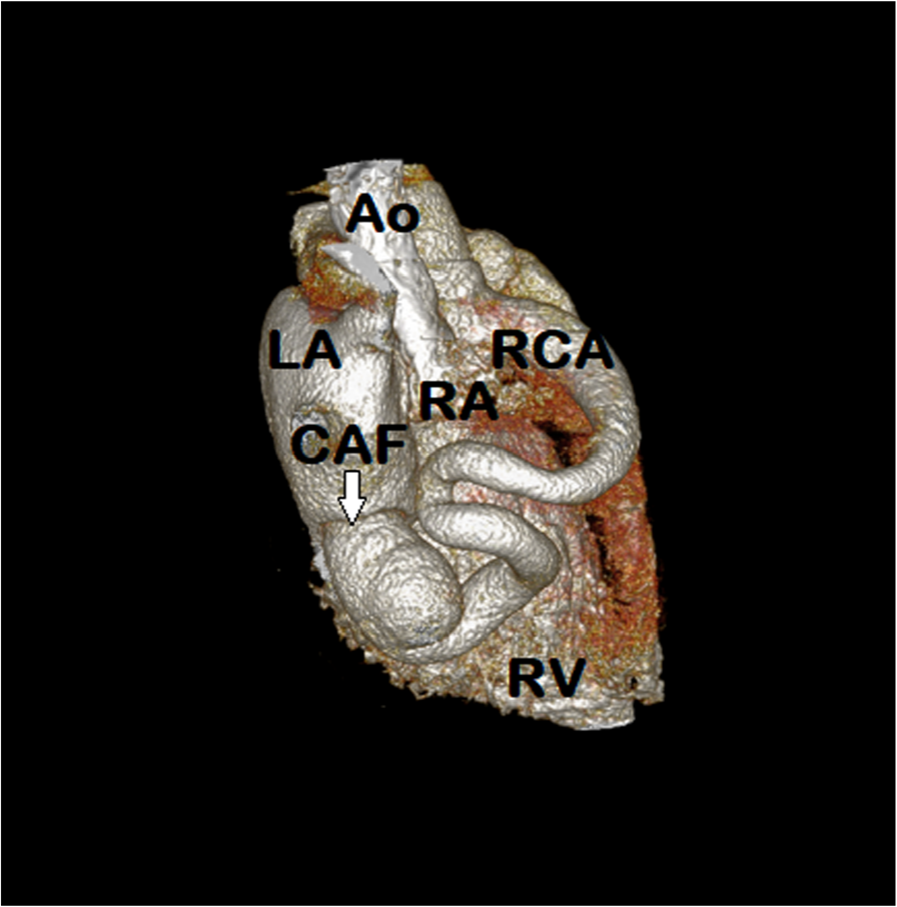
A CT (3D)-reconstruction of the heart showing dilated and tortuous right coronary artery originating from the ascending aorta, traversing through the atrioventricular groove and terminating into the floor of the left atrium. Ao- Ascending aorta, LA-Left atrium, RA- Right atrium, RCA-Right coronary artery, CAF- Coronary artery fistula, RV-Right ventricle

The 2-D echocardiogram including color ‘Doppler’ revealed a dilated left ventricle and left atrium with turbulence in the left atrial cavity. A large cystic mass adjacent to the aortic root with continous flow was seen. The ejection fraction was 50–55%. A coronary angiography done through the right radial approach demonstrated normal left coronary artery with right dominance. The right coronary artery (RCA) was huge in size measuring 17–19 mm in diameter. For a better delineation of the course of the involved coronary artery and relationship of surrounding structures, we performed a computerized tomographic angiography (CTA) of the heart. It revealed normal origin of the coronary arteries. Left coronary artery measured 4.5 mm with normal branching pattern. There was a giant patent RCA with a diameter of 20 mm and a tortuous course. The dilated RCA traversed through the right atrioventricular groove posteriorly and eventually ended into a dilated sac on the posterior wall of the left atrium (Figs. 2 and 3). The dilated sac measured 30 mm in widest dimension and finally entered the floor of the left atrial cavity just above the mitral valve. There was no thrombus or stenosis of the coronary arteries. In addition, there was no evidence of a patent ductus arteriosus.

**Fig. 4:**
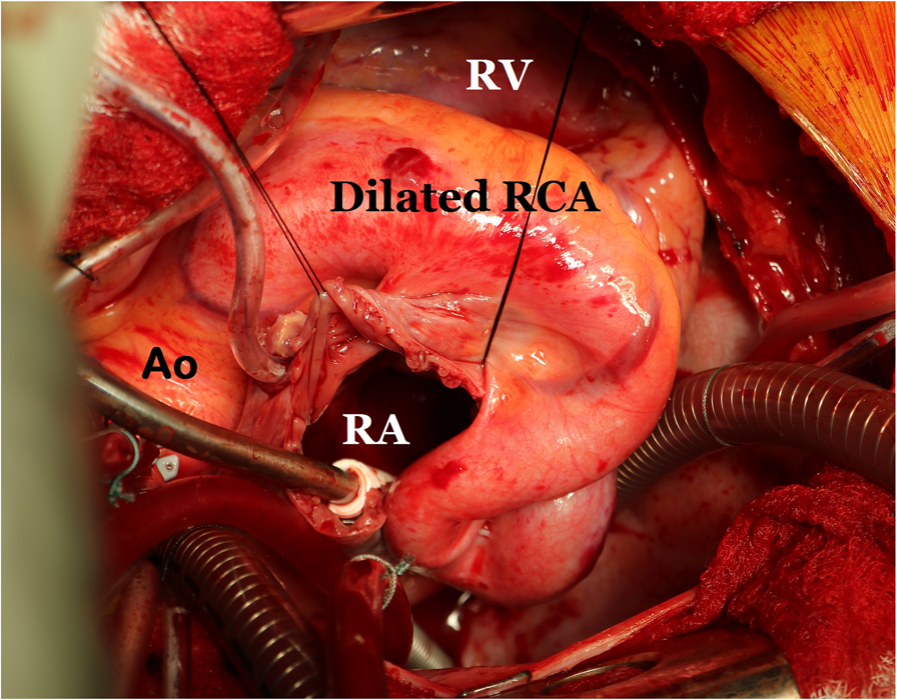
Operative photograph showing fistula tract of the aneurysmal right coronary artery and surrounding structures. RA- Right atrium, Ao- Ascending aorta, RV- Right Ventricle, RCA-Right coronary artery

**Fig. 5:**
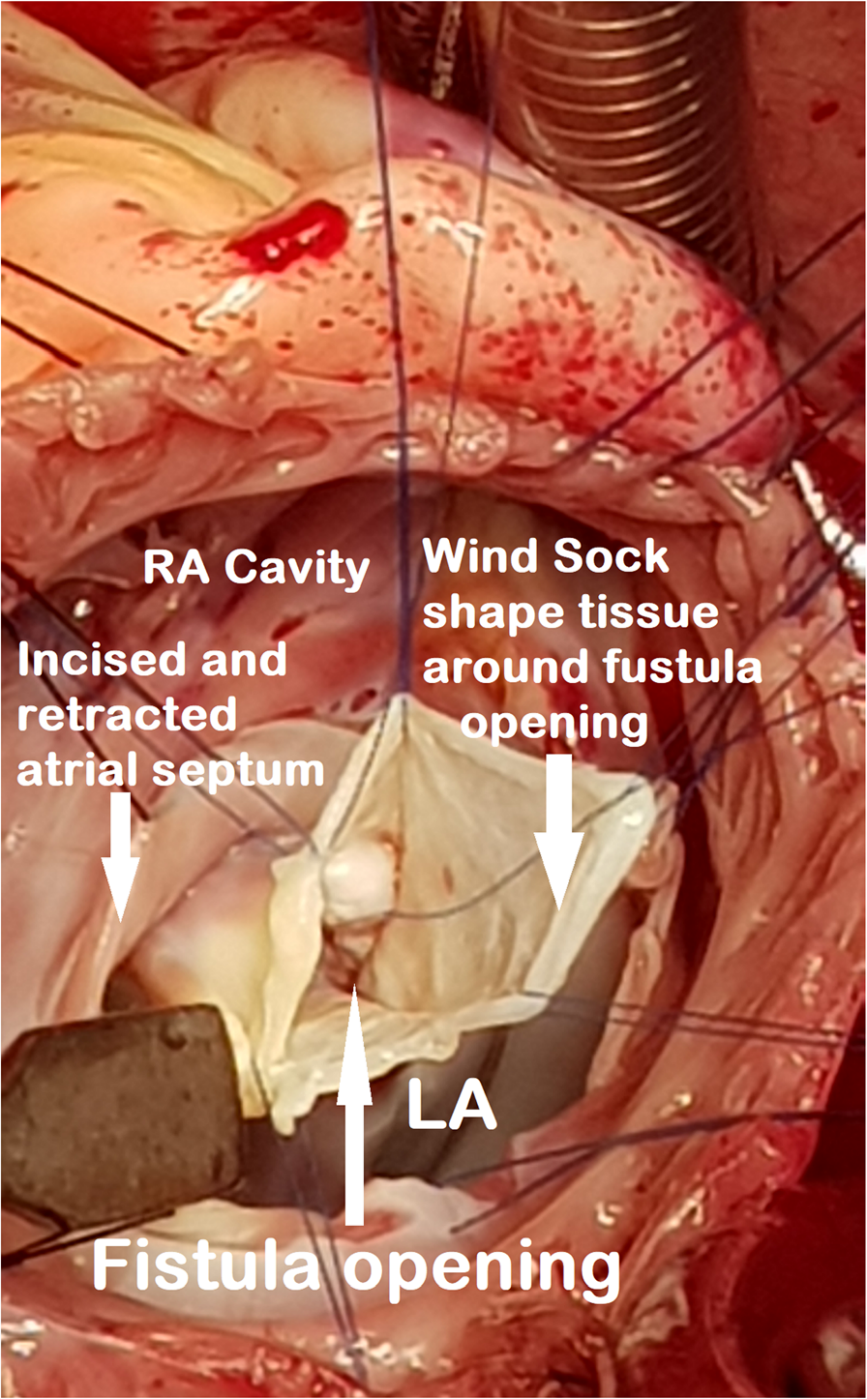
Operative photograph showing opening of the fistula tract of the right coronary artery into the left atrial floor with a ‘windsock’ guarding the orifice of the fistula. RA- Right atrium, LA- Left atrium

In view of recurrent chest discomfort, progressive dyspnea, cardiac enlargement, size of the involved coronary artery and location of the fistulous opening, the patient was considered for a surgical closure under standard cardiopulmonary bypass. The surgery was performed via median sternotomy, bi-caval cannulation, ascending aortic cannulation and aortic cross clamping. Alternating retrograde and antegrade crystalloid cardioplegia technique was used for maximum myocardial protection. The right coronary artery was hugely dilated in its entire course which was long, tortuous with friable surrounding tissue (Fig. 4). Then right atrium was opened which showed no abnormality. Subsequently, the interatrial septum was opened, and the fistula was identified. It was located just above the mitral valve in the floor of the left atrium and the opening (1 cm) was guarded by a membranous windsock shape tissue (Fig. 5). A complete resection of the redundant tissue was done and then the fistulous opening was closed using glutaraldehyde–treated autologous pericardial patch. The atrial septum and the right atrium were closed with running sutures. The weaning from cardiopulmonary bypass was uneventful. There were no ischemic changes and the patient remained in sinus rhythm after the procedure.

There was a good immediate and late postoperative recovery. Currently, (almost 6-months from surgery) the patient is receiving prophylactic aspirin and warfarin to prevent thrombosis or ischemic events. He will receive antibiotic prophylaxis and a careful 6-monthly follow up.

## Discussion

Overall, CAFs have an estimated prevalence of 0.002% in general population, but 0.25–0.5% in patients who undergo coronary angiography [1–7]. There is no race or gender predilection for this entity. In majority of the patients, the CAFs are congenital in origin. During early prenatal life, intra myocardial trabecular sinusoids freely connect the heart cavity with the coronary veins and arteries, but later they close spontaneously. A failure of closure leads to a fistulous communication between coronary arteries and the cardiac chamber [4, 5]. The CAFs are classified into coronary cameral fistulae, where fistula terminates in a cardiac chamber, and arteriovenous fistulae, where the fistula terminates into a venous structure [8]. The ‘Sakakibara’ classification described it as ‘proximal type’ where segment of the involved artery is dilated proximal to the fistula or a ‘distal type’ in which entire coronary artery is dilated in association with a distal fistula. Usually, distal CAFs are difficult to manage and likely to require surgical treatment [6–8].

Low-pressure structures are the most common sites of drainage of the CAF. The usual sites of communications are the right ventricle (41%), right atrium (26%), pulmonary artery (17%), coronary sinus (7%), left atrium (5%), left ventricle (3%), and superior vena cava (1%) [3–6, 8].

Although, majority of adult patients are asymptomatic, approximately 19–63% of the patients start to have symptoms by the age of 18 years [7]. Interestingly, coronary artery aneurysm is found in 17% of the patients with CAFs. [5–7]. Right coronary artery is involved in 50% of these cases. The left coronary artery is the source of CAF in 42% of the patients [1–4]. The clinical presentation of CAFs is mainly dependent on the severity of the left-to-right shunt. The pathophysiology and hemodynamic changes are determined by the size of the communication and the resistance in the chamber of termination. A hemodynamic large shunt flow may result in shunting of flow through the fistula and away from the myocardium that leads to the “coronary steal” phenomenon or ischemia or congestive heart failure [6, 7]. The presence of cardiomegaly and electrocardiographic evidence of left ventricular hypertrophy or ischemia may indicate early overload.

Some patients may present with a complications of CAF such as rupture or thrombosis of the fistula (19–26%), sub-acute bacterial endocarditis (10%) and myocardial infarction (3%) [2–6]. However, the most common features are dyspnea, fatigue, chest pain and orthopnea. The classical finding in these patients is presence of a continuous heart murmur over the precordium [1].

Electrocardiographic findings may show ventricular enlargement, evidence of ischemia or arrhythmias. In our patient, there was evidence of left ventricle enlargement (‘ST’changes) in the precordial leads. The 2-D echo including Doppler usually shows cardiac size, function, dilated arterial sac and turbulence at the termination of fistulous tract. However, the image and full course of the artery is difficult to delineate with this diagnostic tool [6].

A coronary angiography is indicated as conclusive evidence for better visualization and planning the management. The conventional coronary angiography delineates proximal course of the involved coronary arteries and fistulae better. However, the terminal segments and tracts are difficult to visualize due to the fact that dye dilution effect of the contract medium entering into the low pressure chamber reduces the quality and details. Therefore, CTA is a noninvasive and accurate imaging technique for detection of major coronary artery anomalies. It allows us to define details of coronary vessels and relationship of mediastinal structures in order to plan appropriate management [1]. Any further investigations such as a ‘scintigraphy’ was not considered because of the fact that the coronary arteries were found to be patent on angiography and the patient was young.

In patients with angina, subacute bacterial endocarditis, congestive heart failure or signs of cardiac failure, surgical or transcatheter intervention is indicated [5–8]. The aim of intervention is to close the fistula and prevent complications. The surgery is more invasive and requires sternotomy, cardiopulmonary bypass and can prolong the hospital stay. The transcatheter technique is least invasive and less expensive method with better cosmetic results and shorter hospital stay. On the contrary, the complications of such management include embolization of the device, dissection of the vessels or failure of the procedures [2–7].

There are various devices available for closure of CAF which include covered stainless-steel coils, detachable balloons, coils, double umbrella device or ‘Amplatzer’ occluders [5–7]. The large fistula, tortuous tract, aneurysmal tissues and involvement of entire coronary artery course are relative contraindications to the transcatheter management and require surgery [5–7]. A review of the literature suggests that antiplatelet therapy and prophylactic precautions against bacterial endocarditis are recommended in all the patients in the long-term [1–4].

Surgical management and operative planning should depend on the presence of symptoms, concomitant cardiac comorbidities, size and the location of CAFs [2–4]. A careful handling of the aneurysmal coronary tissues, appropriate use of myocardial protection techniques and monitoring for ischemia at all the stages of the surgery is key to success. We used initial retrograde crystalloid cardioplegia delivery through direct cannulation of the coronary sinus ostium, alternating with antegrade delivery of the solution for maximum myocardial protection. A simultaneous coronary artery bypass graft procedure should be performed only if the fistula is challenging or coronary artery disease is present. There are variety of surgical procedures described in the available literature that include ligation of the terminal end of the involved coronary artery, direct closure of the proximal, distal or both ends of the involved coronary artery with coronary revascularization, or patch closure of the fistulous opening using native or synthetic materials [2–4]. The surgery can be performed using ‘on’ or ‘off’ pump techniques depending on the nature and extent of the pathology and co-morbid conditions. We chose to close the fistula at the terminal end (left atrium) on cardiopulmonary bypass in arrested heart. This facilitated a good exposure allowing us to examine details such as course of the aneurysmal right coronary artery, location of the fistula near the mitral valve, proper closure of the main and side channels of the fistulous opening and avoidance of perioperative myocardial infarction.

In the available studies, long-term survival after management of CAF is 93, 74 and 68% at 1, 5 and 15-years, respectively [7]. However, complications may include recurrence, myocardial infarction, tricuspid regurgitation, thromboembolism or death (24%) [7].

## Conclusion

The CAF in association with aneurysmal coronary artery can be managed successfully in adults. It requires differential diagnostic work-up, careful evaluation of fistula size, location and condition of the coronary arteries in order to select suitable therapy. In our opinion, intracardiac surgical closure of the CAF is an appropriate option for the patients with late presentation, larger fistula and aneurysmal coronary arteries and those who are not amenable to transcatheter management. However, long-term follow up is required to evaluate effectiveness of the management, recurrence and late-outcome.

## Abbreviations

CAF: Coronary artery fistula
CTA: Computerized tomographic angiography
NYHA: New York Heart Association
RCA: Right coronary artery
